# The Taxonomy Dictionary: a resource for correct spelling of taxa

**DOI:** 10.1099/acmi.0.000521.v3

**Published:** 2023-05-09

**Authors:** Kristian Bagge

**Affiliations:** ^1^​ Department for Virus and Microbiological Special Diagnostics, Statens Serum Institut, Copenhagen, Denmark; ^2^​ Department of Clinical Microbiology, Copenhagen University Hospital Rigshospitalet, Copenhagen, Denmark

**Keywords:** taxonomy, dictionary

## Abstract

This article describes ‘The Taxonomy Dictionary’, a resource that can enhance the spelling engine of a text editor such as Word, so that it can correctly spell every taxon described and listed in the largest taxonomy databases. It contains around 1.4 million unique words, and once installed an incorrectly spelled taxon will be marked by the spelling engine and it will suggest possible correct spellings. Installation instructions for Firefox, LibreOffice and Microsoft Word can be found on the GitHub repository. The software is licensed with a GPL3 licence.

## Data Summary

The databases used for the generation of the dictionary are publicly available; links to the individual databases are listed on the repository [1]. An overview of the databases and which versions are used can be found in Table 1. Installation instructions of the dictionary for Hunspell [2] on Linux, Mozilla Firefox [3], LibreOffice [4] and Microsoft Word [5] can be found on the GitHub repository [1]. It is licensed under a GPL3 licence, which ensures that it and any derivatives are and always will be free for every one to use. For how the data were processed to generate the final result see ‘Construction of the dictionary’ below. The software repository on GitHub [1] contains a script with the steps that were used to create the dictionary. Thereby users can reproduce it, or copy the idea and make their own dictionary on a subject of their interest.

**Table 1.** Version of databases used for construction of The Taxonomy Dictionary v.1.0.3

	Version
GBIF Backbone	23 November 2022
Catalogue of Life	19 December 2022
LPSN	26 January 2023
ICTV	1 November 2022
MycoBank	23 March 2022
Zoobank	9 January 2023
The World Flora Online	28 June 2022

**Impact Statement**

Enumeration of taxa is frequent and abundant in some divisions of biology. Until now, there has existed no dictionary capable of spellchecking every taxonomic name in a document. This resource fills in that gap, and since it contains all taxa enlisted in the largest public databases it will have broad impact, and can hopefully facilitate the writing process for many biologists. For microbiologists the broad scope means that both the infecting agent, its vector and host can be spellchecked.

## Introduction

This article presents a newly created resource, a digital dictionary capable of enhancing the spelling engine of a text editor, so it can spellcheck every taxonomic name in a document. Most people working with biology frequently encounter words or technical terms that have to be manually appended to the local user dictionary. This imposes the risk of incorrectly adding a mistyped word, and ever forward being misled on how to spell that word, until that error might subsequently be corrected. Another aspect is the sheer number of words that have to be added manually. Any automation of this process has the potential to save many hours of work and reduce spelling errors. The present resource was created to fill in that gap, and should be of value for any one working in an area of biology, especially where numbers of taxa are abundant and diverse.

## Construction of the dictionary

The Taxonomy Dictionary consists of a wordlist of around 1.4 million unique words. The wordlist has been generated by aggregating some of the largest taxonomy databases. The major ones are GBIF backbone [6] and Catalogue of Life (COL) [7]. Neither of these is complete, but they both aspire to contain every taxon described worldwide. To ascertain good coverage of microbes, taxonomy databases for viruses, bacteria and fungi have been added, namely the International Committee on Taxonomy of Viruses (ICTV) [8], List of Prokaryotic names with Standing in Nomenclature (LPSN) [9] and MycoBank [10]. For animals and plants Zoobank [11] and World Flora Online (WFO) [12] are used. Table 1 shows the version of each database. The data have been processed so that all metadata have been filtered out, and only the taxon names from each database are kept. The commands are linked together with a Bash [13] shell script available on the Github repository (wordlist_script.sh). The databases are downloaded with curl [14] or wget [15]. For the LPSN database the user can download it using their API. This is handled in a Python [16] script (lpsnapi.py) that is called from the Bash script. Please be aware that the credentials in the python script need to be updated before the script can run (it is free to register on their site). The zip-compressed databases are unzipped. Some databases are downloaded as Excel files. The Excel files are exported to a comma-separated file (CSV) by another Python script (csvexport.py). Then, cut [17] is used to extract the relevant columns containing information on taxa. For a dictionary to be readable by most text editors it must be presented as a long list of words, where each word is presented on a new line. Therefore, sed [18] is used to substitute tabs, commas and spaces with new lines and the outputs are redirected to a separate text file for each database. The text files are concatenated together with cat [19] and are then piped through sort [20] and duplicates are filtered out with uniq [21]. Finally, any words containing numbers or special characters are filtered out with sed, as they are not considered genuine entries. The result is a wordlist containing 1 400 952 unique words.

## Functionality

Installation instructions for Firefox, LibreOffice and Microsoft Word can be found on the GitHub repository. After installation, an incorrectly spelled taxon will be marked by the spelling engine and it will suggest possible correct spellings. However, it will not check if italics are used correctly, so users should be aware of that. In most text editors it is possible to enable it alongside the preferred language. The dictionary is huge, but on any modern computer there should be no performance issues. Due to the size of the dictionary, the autosuggestions may be subject to error; this depends on the spelling engine and can occur if the user tries to spell a word for which there are many similar ones in the dictionary. Most of the time, however, it will suggest a reasonable correction.

If the unlikely should happen and the user finds a taxon not included or has a suggestion for a relevant database to add, they should please reach out, and I will try to incorporate it. I plan to update the dictionary 2–4 times a year depending on if there are any major releases to one of the databases upon which the dictionary is built.

## References

[1] Bagge K (2022). Kbagge/taxonomy_dictionary: taxonomy dictionary. Zenodo.

[2] Ooms J (2023). hunspell: High-performance stemmer, tokenizer,and spell checker.

[3] Mozilla Foundation (2023). Firefox.

[4] The Document Foundation (2023). LibreOffice.

[5] Word (2023). Microsoft.

[6] Registry-Migration.Gbif.Org (2021). GBIF Backbone Taxonomy.

[7] Bánki O, Roskov Y, Döring M, Ower G, Vandepitte L (2022). Catalogue of life checklist (version 2022-03-21). Cat Life.

[8] Lefkowitz EJ, Dempsey DM, Hendrickson RC, Orton RJ, Siddell SG (2018). Virus taxonomy: the database of the International Committee on Taxonomy of Viruses (ICTV). Nucleic Acids Res.

[9] Parte AC, Sardà Carbasse J, Meier-Kolthoff JP, Reimer LC, Göker M (2020). List of Prokaryotic names with Standing in Nomenclature (LPSN) moves to the DSMZ. Int J Syst Evol Microbiol.

[10] Robert V, Vu D, Amor ABH, van de Wiele N, Brouwer C (2013). MycoBank gearing up for new horizons. IMA Fungus.

[11] Pyle R (2022). ZooBank.

[12] WFO (2022). World Flora Online. Version 2022.7. Published onthe Internet. http://www.worldfloraonline.org.

[13] GNU P (2007). Free software foundation. Bash (3.2. 48)[Unixshell program].

[14] Hostetter M, Kranz DA, Seed C, Terman C, Ward S (1997). Curl: a gentle slope language for the web. World Wide Web J.

[15] Niksic H (2015). Free software foundation. WGET (1.21.3).

[16] Van Rossum G, Drake FL (1995). Python reference manual.

[17] GNU P Free software foundation. cut (GNU core utils).

[18] GNU P Free software foundation. GNU sed.

[19] GNU P Free software foundation. cat (GNU core utils).

[20] GNU P Free software foundation. sort (GNU core utils).

[21] GNU P Free software foundation. uniq (GNU core utils).

